# A multicenter performance evaluation of cefiderocol MIC results: ComASP in comparison to CLSI broth microdilution

**DOI:** 10.1128/jcm.00926-24

**Published:** 2024-12-31

**Authors:** L. M. Koeth, J. M. DiFranco-Fisher, E. Palavecino, A. Kilic, D. Hardy, D. Vicino, S. Stracquadanio, S. Stefani

**Affiliations:** 1 Laboratory Specialists, Inc., Westlake, Ohio, USA; 2 School of Medicine, Wake Forest University 12279 , Winston-Salem, North Carolina, USA; 3 Clinical Microbiology, University of Rochester Medical Center 6923 , Rochester, New York, USA; 4 Clinical Microbiology, University of Catania 60279 , Catania, Italy; University of Calgary, Calgary, Canada

**Keywords:** ComASP Cefiderocol, broth microdilution, multicenter study

## Abstract

The performance of the Liofilchem Compact Antimicrobial Susceptibility Panel (ComASP) Cefiderocol was evaluated in a multicenter study. Enterobacterales, *Acinetobacter baumannii*, and *Pseudomonas aeruginosa* clinical isolates and challenge isolates were tested by three and one sites, respectively. Minimum inhibitory concentration (MIC) testing was performed by the Clinical and Laboratory Standards Institute (CLSI) broth microdilution and ComASP, which included two reading endpoints (CLSI read; MIC is the first well in which reduction of growth is <1 mm or light haze/faint turbidity] and ComASP [ComASP read; MIC is the first well at which 100% inhibition of growth occurs]). Each site performed reproducibility and quality control (QC) by ComASP and broth microdilution (BMD). Reproducibility was excellent (97.4% within ±1 dilution of modal MIC). All QC results were within CLSI QC ranges by BMD and ComASP, except for two *E. coli* ATCC 25922 results from one site. Essential agreement for combined clinical and challenge Enterobacterales was 84.3% (CLSI read) and 95.7% (ComASP read), *P. aeruginosa* was 83.3% (CLSI read) and 93.7% (ComASP read), and *A. baumannii* was 78.3% (CLSI read) and 96.7% (ComASP read). Categorical agreement for Enterobacterales was 92.4% for both CLSI read and ComASP read, for *P. aeruginosa* was 89.7% (CLSI read) and 92.1% (ComASP read), and for *A. baumannii* was 72.8% (CLSI read) and 91.3% (ComASP read). There were no very major errors using the ComASP read. One very major error for *P. aeruginosa* occurred using the CLSI read method. Three very major errors for *A. baumannii* occurred using the CLSI read method. ComASP Cefiderocol was shown to be a reliable method for testing cefiderocol MIC against relevant clinical isolates when ComASP read is used.

**IMPORTANCE:**

There are very limited commercial methods available to clinical laboratories for cefiderocol minimum inhibitory concentration (MIC) testing. The Compact Antimicrobial Susceptibility Panel (ComASP) Cefiderocol method includes iron-depleted cation-adjusted Mueller–Hinton broth, which eliminates variability in cefiderocol MIC results based on iron levels. The lyophilized multi-well format of ComASP also provides for room temperature storage. In comparison to what an individual lab may do for method verification, this multi-site, multi-isolate study provides a robust evaluation and greater assurance to clinical microbiologists of the method's accurate and reproducible performance.

## INTRODUCTION

Cefiderocol is a siderophore-conjugated cephalosporin antibacterial agent indicated in the United States for patients 18 years of age or older for the treatment of complicated urinary tract infections (cUTI), including pyelonephritis, and hospital-acquired bacterial pneumonia and ventilator-associated bacterial pneumonia (HABP/VABP) (1). The siderophore component of cefiderocol binds iron and uses the bacterial iron transport system for drug entry into the bacterial periplasmic space, where the iron atom disassociates from the siderophore, crosses the cytoplasmic membrane into the cytoplasm, leaving cefiderocol in the periplasmic space. Once in the periplasmic space, cefiderocol is able to inhibit bacterial cell wall synthesis by binding to penicillin-binding proteins and inhibiting peptidoglycan synthesis, which results in cell death (2). Recent surveillance studies demonstrated that cefiderocol was highly active *in vitro* against Enterobacterales, *Pseudomonas aeruginosa*, and *Acinetobacter* species isolates carrying metallo-β-lactamases and serine carbapenemases, as well as carbapenemase-negative, and meropenem-nonsusceptible isolates (3). Cefiderocol also has *in vitro* activity against *Stenotrophomonas maltophilia* (4).

Because bacterial iron transporters are upregulated under iron-depleted conditions such as those found *in vivo*, but not in the iron concentrations found in standard cation-adjusted Mueller–Hinton broth (CA-MHB), a modification of the reference broth microdilution (BMD) is required for cefiderocol susceptibility testing. This modified method uses iron-depleted CA-MHB to a final iron concentration of ≤0.03 µg/mL, which requires special preparation by the laboratorian (5). Given the high vacancy rate in US clinical microbiology laboratories and the time and effort to prepare iron-depleted CA-MHB, a testing device that both demonstrates high performance and does not require the preparation of iron-depleted CA-MHB should be well received by clinical microbiologists (6).

The ComASP Cefiderocol (Compact Antimicrobial Susceptibility Panel, Liofilchem, Italy) is a Food and Drug Administration (FDA)-cleared quantitative BMD method for the *in vitro* determination of antimicrobial susceptibility of *Acinetobacter baumannii* complex, *Escherichia coli*, *Enterobacter cloacae* complex, *Klebsiella pneumoniae*, *Proteus mirabilis*, *P. aeruginosa*, and *Serratia marcescens*. Each ComASP Cefiderocol panel includes cefiderocol across 15 twofold dilutions from 0.008 to 128 µg/mL and a positive control well in lyophilized form in two rows and the same wells in the next two rows, allowing for testing of two bacterial isolates per panel which can include a quality control strain. All wells are rehydrated with a standardized microbial suspension made in the iron-depleted CA-MHB broth, which is provided in the kit. After incubation in ambient air for 16–20 h, the minimum inhibitory concentration (MIC) result is read at the lowest concentration that completely inhibits visible growth (7). The primary objective of this paper is to describe a multicenter study comparing the ComASP Cefiderocol to Clinical and Laboratory Standards Institute (CLSI) modified reference broth microdilution for submission to FDA for device clearance. A secondary objective of this paper is to report the results of two reading methods employed for ComASP in the study as follows: (i) the CLSI recommended cefiderocol MIC reading method and (ii) the ComASP Cefiderocol reading method (100% inhibition of growth as the MIC endpoint) (5, 7).

## MATERIALS AND METHODS

The study design and analysis were in accordance with the FDA, Center for Device and Radiological Health (CDRH), antimicrobial susceptibility methods guidance document (8). Clinical, reproducibility, and quality control (QC) isolates were tested at the following two sites by ComASP: (i) Wake Forest Baptist Medical Center, Winston—Salem, NC, USA, and (ii) University of Rochester Medical Center, Rochester, NY, USA. Clinical and QC isolates were tested by ComASP at the University of Catania, Catania, Italy, and reproducibility and QC isolates only were tested at Liofilchem S.r.l, Roseto degli Abruzzi, Italy. Laboratory Specialists, Inc., Westlake, OH, USA, performed testing of reference BMD for all clinical isolates and both reference BMD and ComASP (using the same initial inoculum) for challenge isolates (Table S1).

### Study isolates

All three sites tested 20 replicates of two QC strains (*E. coli* ATCC 25922 and *P. aeruginosa* ATCC 27853). A set of 10 reproducibility isolates included the clinically indicated species with a range of MIC results from 0.12 to 32 µg/mL (2 *A*. *baumannii*, 1 *E. coli*, 2 *K*. *pneumoniae*, 1 *P*. *mirabilis*, 1 *S*. *marcescens,* and 3 *P*. *aeruginosa*). Included in the study was a three-site collection of 303 clinical isolates (for the clinically indicated species), which encompassed Enterobacterales (30 *E. cloacae*, 33 *E. coli*, 30 *K. pneumoniae*, 24 *P*. *mirabilis,* and 21 *S*. *marcescen*), 75 *A*. *baumannii,* and 90 *P*. *aeruginosa*. A total of 61.7% of these clinical isolates were tested within 6 months of collection. In addition, 126 isolates, which include non-clinically indicated species, were also tested using the ComASP Cefiderocol and compared to broth microdilution (12 *Citrobacter freundii*), 13 *Citrobacter koseri*, 12 *Klebsiella aerogenes*, 13 *Klebsiella oxytoca*, 12 *Morganella morganii*, 12 *Providencia rettgeri*, 12 *Proteus vulgaris,* and 40 *S*. *maltophilia*. A “challenge” set of 125 isolates were also tested with the majority having elevated cefiderocol MIC results (63.2% of reference MIC results were 4- > 256 µg/mL), and molecular characterization was available for 44% of isolates (12 *E. cloacae*; 22 *E. coli;* 20 *K. pneumoniae*; 10 *P*. *mirabilis* and eight *S*. *marcescens*, 17 *A*. *baumannii* and 36 *P*. *aeruginosa* [Table S2]).

### MIC methods

**Fig 1:**
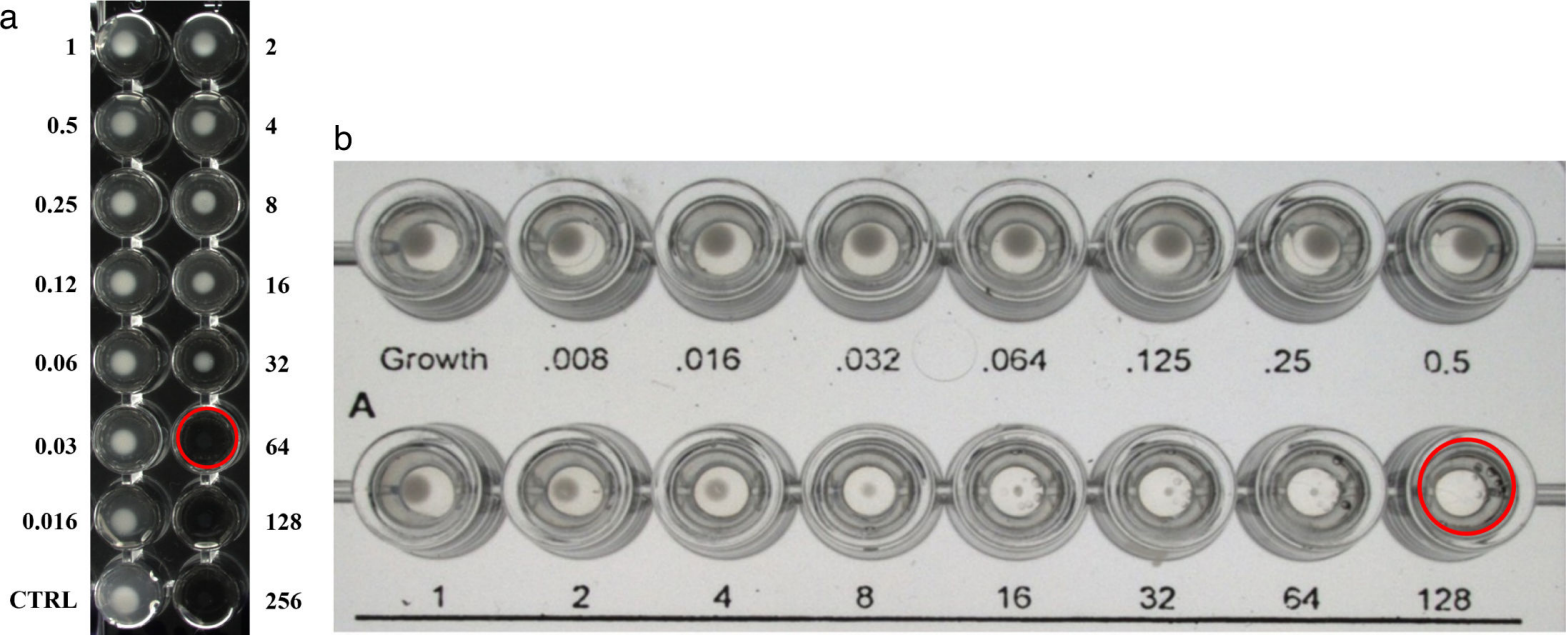
Example picture of *A. baumannii* read by reference BMD and ComASP (red circle indicates MIC) (a) Reference BMD: Cefiderocol MIC = 64 µg/mL, (b) ComASP: Cefiderocol MIC = 128 µg/ml (MIC by CLSI read = 8 µg/mL).

Manufacturer’s procedures were used for testing the ComASP Cefiderocol (using one lot) with the exception of the addition of a second reading method (5). All sites inoculated the panels with a single or multichannel pipet, sealed the panels with the seal-provided incubated panels at 36 ± 2°C for 16–20 h in ambient air, stacked the panels no more than four high, and read the panels manually, using bright indirect lighting against a dark background to improve reading if needed. The two reading endpoints that were evaluated for ComASP in the 510(k) study were as follows: (i) the CLSI cefiderocol reading recommendation for those isolates without a clear endpoint (designated “CLSI read”), which states “the MIC is read as the first well in which reduction of growth corresponds to <1 mm or is replaced by the presence of light haze/faint turbidity” and (ii) the ComASP Cefiderocol reading method (designated “ComASP read”) in which the MIC is read at the first well at which 100% inhibition of growth occurs) (5). An example picture of MIC reads for reference BMD and both ComASP reads is shown in Fig. 1. The CLSI reference methodology was followed utilizing a single lot of frozen broth microdilution panels containing iron-depleted CAMHB (prepared with a single lot of BBL brand CAMHB, Becton Dickinson, Sparks, MD, USA, and cefiderocol powder provided by Shionogi). All reference BMD testing was based on one reading method (CLSI). The iron level in the iron-depleted CAMHB was <0.01 µg/mL (Visocolor HE Colorimetric test, Macherey-Nagel, Allentown, PA), and in addition to the routine quality control strains, the cefiderocol MIC was determined and within the established range for an internal control strain (*P. aeruginosa* SR27001, cefiderocol MIC range 0.5–4 µg/mL) (5, 9). All replicate testing of the QC strains was performed with the ComASP method and reference BMD method, and results were compared to the CLSI expected ranges (5). Colony counts were performed from the positive growth control of the ComASP panel. This included one replicate of each reproducibility isolate, all QC replicates, and at least 10% of the clinical isolates tested. Purity checks were performed on all QC, reproducibility, and clinical isolates using the ComASP panel growth control.

### Data analysis

Essential agreement (EA) was calculated based on the percentage of ComASP results that were within one dilution of the BMD results. Categorical agreement (CA) and errors were based on FDA cefiderocol susceptibility interpretative criteria (10). Very major errors (VME) were defined as isolates resistant or non-susceptible by BMD MIC and susceptible by ComASP MIC. Major errors (ME) were defined as isolates susceptible by BMD MIC and resistant by ComASP MIC. Minor errors (mE) were defined as isolates susceptible by BMD MIC and intermediate by ComASP MIC, intermediate by BMD MIC and susceptible by ComASP MIC, intermediate by BMD MIC and resistant by ComASP MIC, and resistant by BMD MIC and intermediate by ComASP MIC. VME percents were based on the number of very major errors divided by the number of resistant isolates, and ME percents were based on number of major errors divided by the number of susceptible isolates. All ComASP results were compared for the determination of any trending (i.e., lower or higher results) compared to the reference method. Isolates with off-scale MIC results by both methods were excluded from this trending analysis. The total number of results ≥1 dilution lower and ≥1 dilution higher than the reference MIC was determined. The percentage of results ≥1 dilution lower was subtracted from the percentage of results ≥1 dilution higher and divided by the total number of isolates with on-scale MIC results for determination of the overall trending percents. All reproducibility results were compared to the overall modal MIC result for each strain.

Acceptance criteria based on the FDA guidance document were (i) EA and CA >89.9%. It is noteworthy that a CA of <90% may be acceptable under certain circumstances (e.g., very good EA of the evaluable test results with the majority of the discrepancies as minor discrepancies). (ii) ME <3% based on the number of susceptible organisms tested. (iii) VME <1.5% based on the number of resistant organisms tested. (4) A ≥30% trending percent is considered noteworthy for the device label (which is based on recent FDA CDRH 510(k) review guidance and not included in the current guidance document). (v) At least 95% of reproducibility results must be within ±1 dilution of the modal MIC results.

## RESULTS

Quality control and reproducibility summary results are shown in Tables 1 and 2. QC results were within established CLSI QC ranges for all isolates by BMD and ComASP, except for two *E. coli* ATCC 25922 with ComASP results of 1 µg/mL from one site on 1 day.

**TABLE 1:** Cefiderocol BMD and ComASP MIC results (*n*) for quality control strains (ComASP reading method)^*a*^

QC organism	Expected result	MIC	Reference frequency (LSI)	ComASP frequency					
		µg/mL		Site 1	Site 2	Site 3	LSI	Liofilchem	All sites
*E. coli* ATCC 25922	0.06–0.5	0.03							
		0.06	5						
		0.12	18	14	18		3	11	46
		0.25	3	6	2	17	3	2	30
		0.5				3			3
		1				2^*b*^			2^*b*^
*P. aeruginosa* ATCC 2785*3*	0.06–0.5	0.03							
		0.06		8				4	12
		0.12	7	12	19	11		9	58
		0.25	16		1	9	3		29
		0.5	2			2	2		6
		1							

^*a*^ Shaded area indicates CLSI-established quality control ranges.

^*b*^ Both results were from two replicates tested on the same day, and no data, other than quality control results, were reported on this day. All subsequent results were within the expected CLSI range.

**TABLE 2:** Summary of cefiderocol ComASP reproducibility results by organism (ComASP reading method)

Reproducibility strain no., Species	Difference in the number of doubling dilutions between new test result and test mode								Mode
	Off-Scale	−2	−1	0	1	2	>2	Off-scale	
All sites									
R1, *A. baumannii*			8	16	3				0.5
R2, *E. coli*		1	13	13					1
R3, *K. pneumoniae*				13	13	1			0.12
R4, *K. pneumoniae*			2	23	2				4
R5, *P. aeruginosa*			12	13	2				0.12
R6, *P. aeruginosa*			2	18	7				4
R7, *P. aeruginosa*			10	15	2				16
R8, *P. mirabilis*			4	12	6	5			0.12
R9*, A. baumannii*			5	18	4				32
R10, *S. marcescens*			10	16	1				16
Total		1	66	157	40	6			
Between-site reproducibility			263/270 = 97.4%						

Inter- and intra-laboratory precision was high for the Enterobacterales, *Acinetobacter baumannii* and *P. aeruginosa* reproducibility isolates; 97.4% of the results were within one dilution of the modal MIC for each organism (Table 2).

### Clinical and challenge set results

#### Enterobacterales

**Fig 2:**
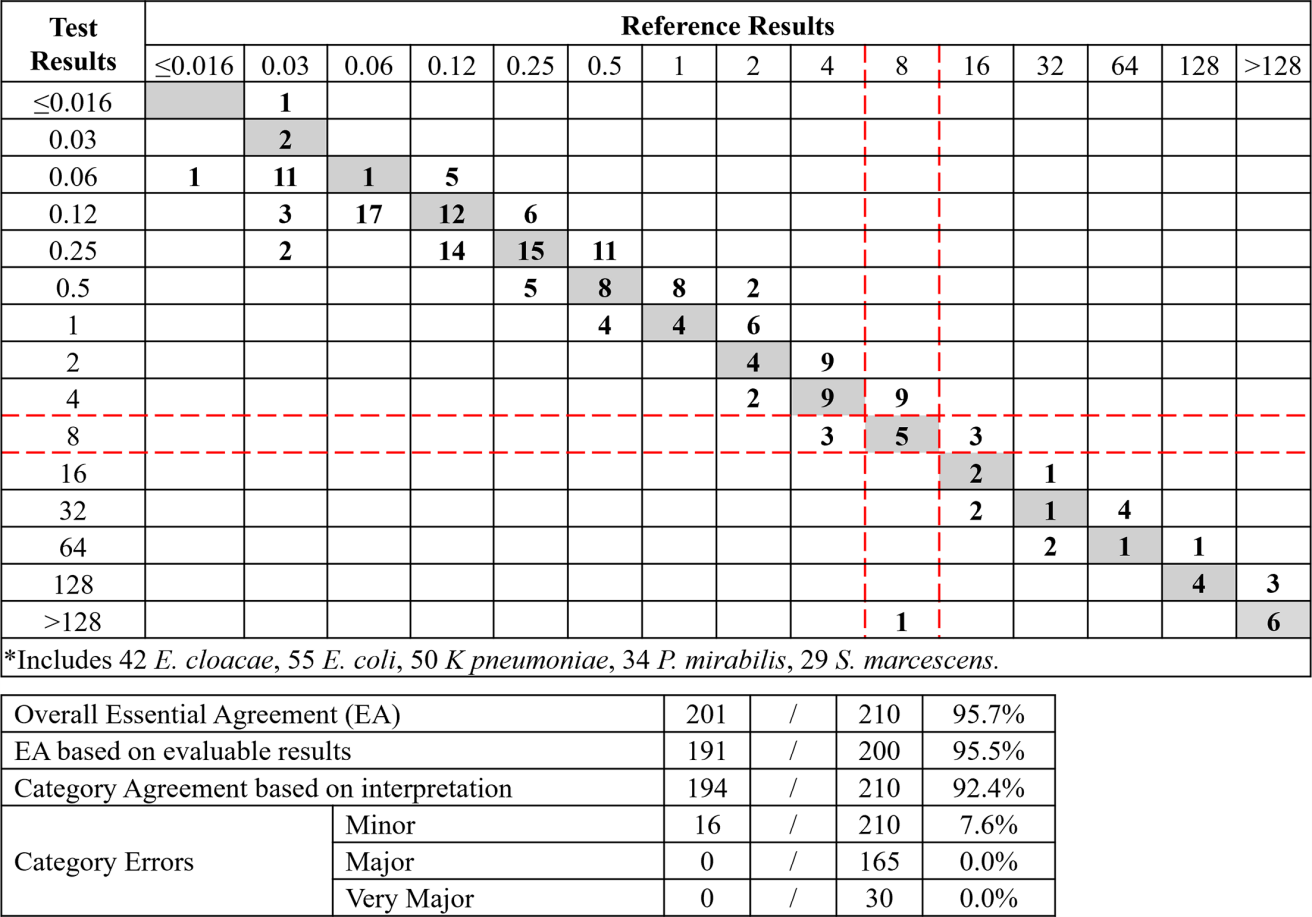
Comparison of ComASP MIC distribution to BMD MIC distribution for 210 Enterobacterales* (combined clinical and challenge isolates; ComASP reading method; FDA/CLSI Breakpoints [indicated with red-dotted lines]).

EA for combined clinical and challenge Enterobacterales was 84.3% for CLSI read and 95.7% for ComASP read. Categorical agreement for Enterobacterales was 92.4% for both CLSI read and ComASP read (Table 3; Fig. 2). Among the individual species of Enterobacterales, EA for CLSI /ComASP reads for the combined clinical and challenge isolates were 83.3%/97.6% for *E. cloacae*, 89.1%/94.5% for *E. coli*, 82.0%/96.0% for *K. pneumoniae*, 76.5%/91.2% for *P. mirabilis* and 89.7%/100% for *S. marcescens*. The categorical agreement for CLSI /ComASP reads for the combined clinical and challenge isolates were 85.7.1%/85.7% for *E. cloacae*, 94.5%/94.5% for *E. coli*, 90.0%/90.0% for *K. pneumoniae*, 100%/97.1% for *P. mirabilis*, and 93.1%/96.6% for *S. marcescens*. No VME was using the ComASP read method, and one VME for *E. cloacae* occurred using the CLSI read method. Trending of higher ComASP (ComASP read) compared to BMD results was observed with *P. mirabilis* (31 trending percent). Trending of lower ComASP (CLSI read) compared to BMD results was observed with *E. cloacae* (−50.0 trending percent). The mean colony counts (CFU/mL) for the ComASP for clinical and challenge Enterobacterales isolate testing were 4.98 × 10^5^ and 5.84 × 10^5^, respectively, and for BMD, 5.46 × 10^5^ and 4.79 × 10^5^, respectively.

**TABLE 3:** Summary of ComASP using 2 different reading methods compared to CLSI BMD MIC by bacterial species for combined clinical and challenge organisms (category agreement and errors based on FDA breakpoints)^*a*^^,^^*b*^

Organism group	Total tested	#EA	%EA	#CA	%CA	#R	#VME	#ME	#mE
ComASP results based on CLSI read (ignoring faint growth)									
*E. cloacae*	42	35	83.3%	6	85.7%	4	1	0	5
*E. coli*	55	49	89.1%	52	94.5%	9	0	0	3
*K. pneumoniae*	50	41	82.0%	45	90.0%	8	0	0	5
*P. mirabilis*	34	26	76.5%	34	100%	6	0	0	0
*S. marcescens*	29	26	89.7%	27	93.1%	3	0	0	2
Enterobacterales	210	177	84.3%	194	92.4%	30	1	0	15
*P. aeruginosa*	126	105	83.3%	113	89.7%	16	1	0	12
*A. baumannii*	92	72	78.3%	67	72.8%	39	3	0	22
Total	428	354	82.7%	374	87.4%	85	5	0	49
ComASP results based on ComASP read (100% inhibition)									
*E. cloacae*	42	41	97.6%	36	85.7%	4	0	0	6
*E. coli*	55	52	94.5%	52	94.5%	9	0	0	3
*K. pneumoniae*	50	48	96.0%	45	90.0%	8	0	0	5
*P. mirabilis*	34	31	91.2%	33	97.1%	6	0	0	1
*S. marcescens*	29	29	100%	28	96.6%	3	0	0	1
Enterobacterales	210	201	95.7%	194	92.4%	30	0	0	16
*P. aeruginosa*	126	118	93.7%	116	92.1%	16	0	0	10
*A. baumannii*	92	89	96.7%	84	91.3%	39	0	0	8
Total	428	408	95.3%	394	92.1%	85	0	0	34

^*a*^ EA, essential agreement; CA, category agreement; R, resistant; VME, very major error; ME, major error; mE, minor error.

^*b*^ The grey shades indicates that the MIC is the same by both methods.

#### Pseudomonas aeruginosa

**Fig 3:**
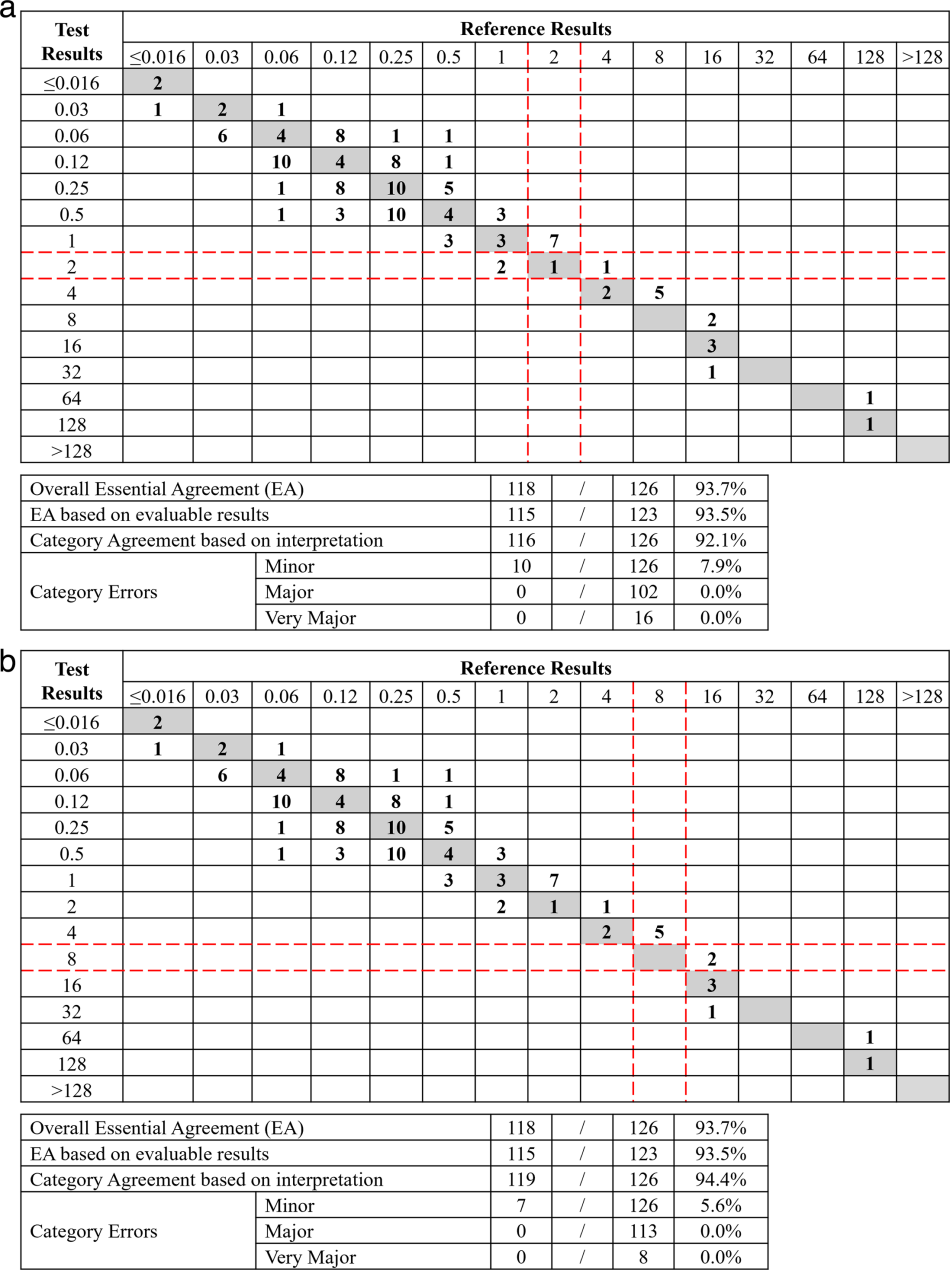
Comparison of ComASP MIC distribution to BMD MIC distribution for 126 *P*. *aeruginosa* (combined clinical and challenge isolates; ComASP reading method) (a) FDA Breakpoints (indicated with red lines). (b) CLSI Breakpoints (indicated with red-dotted lines).

EA for combined clinical and challenge *P. aeruginosa* was 83.3% for CLSI read and 93.7% for ComASP read (Table 3; Fig. 3). Categorical agreement for *P. aeruginosa* was 89.7% for CLSI read and 92.1% for ComASP read. There were no very major errors using the ComASP read method, and one very major error for *P. aeruginosa* occurred using the CLSI read method. Trending was not observed for this organism using both reading methods. The mean colony counts (CFU/mL) for the ComASP for clinical and challenge *P. aeruginosa* testing were 5.4 × 10^5^ and 4.85 × 10^5^, respectively, and for BMD, 5.96 × 10^5^ and 3.97 × 10^5^, respectively.

#### Acinetobacter baumannii

**Fig 4:**
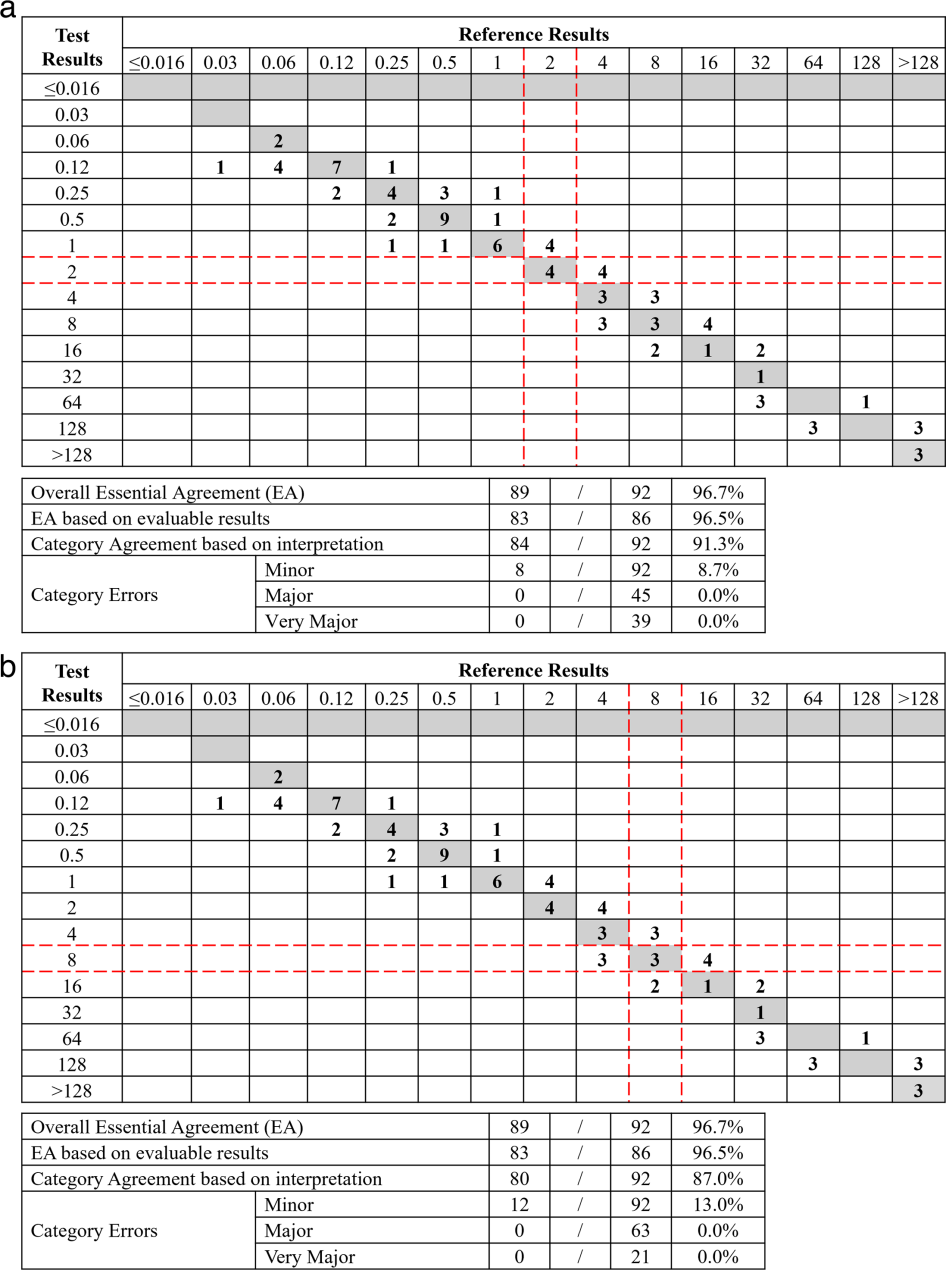
Comparison of ComASP MIC distribution to BMD MIC distribution for 92 *A*. *baumannii* (combined clinical and challenge isolates; ComASP reading method) (a) FDA Breakpoints (indicated with red lines). (b) CLSI Breakpoints (indicated with red-dotted lines).

EA for combined clinical and challenge *A. baumannii* were 78.3% for CLSI read and 96.7% for ComASP read (Table 3; Fig. 4). CA for *A. baumannii* was 72.8% for CLSI read and 91.3% for ComASP read. No VME was using the ComASP read method, and three VME for *A. baumannii* occurred using the CLSI read method. Trending (ComASP read) was not observed for this organism. Trending of lower ComASP (CLSI read) compared to BMD results was observed with *E. cloacae* (−50.6 trending percent). The mean colony counts (CFU/mL) for the ComASP for clinical and challenge *A. baumannii* isolate testing were 4.91 × 10^5^ and 4.77 × 10^5^, respectively, and for BMD, 4.32 × 10^5^ and 5.08 × 10^5^, respectively.

### Non-Clinically indicated species results

#### Stenotrophomonas maltophilia

**Fig 5:**
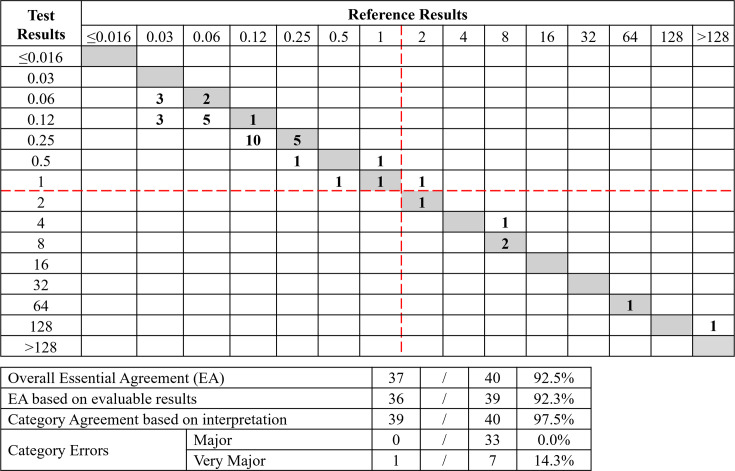
Comparison of ComASP MIC distribution to BMD MIC distribution for 40 *S*. *maltophilia* (combined clinical and challenge isolates; ComASP reading method; CLSI breakpoint [indicated with red-dotted line]).

EA for combined clinical *S. maltophilia* isolates was 82.5% for CLSI read and 92.5% for ComASP read. One VME for *S. maltophilia* (CLSI breakpoint) occurred resulting in a CA of 97.5% for ComASP read. (Fig. 5). Trending of higher ComASP (ComASP read) compared to BMD results was observed with *S. maltophilia* (47.5 trending percent). Trending of ComASP (CLSI read) compared to BMD results was not observed with *S. maltophilia*.

### Enterobacterales

**Fig 6:**
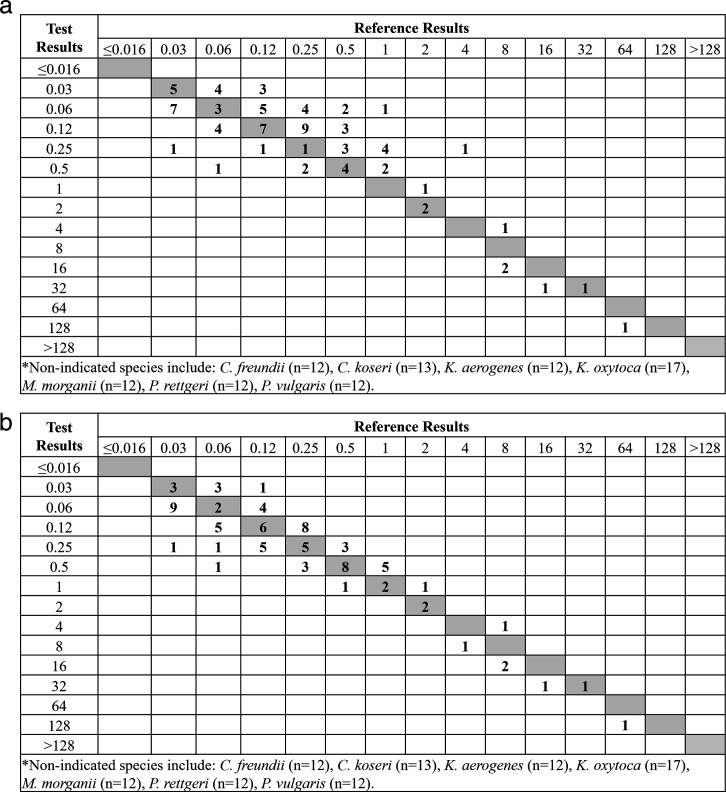
Comparison of ComASP MIC distribution to BMD MIC distribution for Enterobacterales species that are not FDA clinically indicated (combined clinical and challenge isolates) (a) Consolidated Enterobacterales (non-clinically indicated species)*, CLSI Read, EA = 76.7% (b) Consolidated Enterobacterales (non-clinically indicated species)*, ComASP Read, EA = 95.3%.

EA for combined clinical non-indicated species was 76.7% for CLSI read and 95.3% for ComASP read (Fig. 6). (Fig. S1; Fig. 2) Trending of lower ComASP (CLSI read) compared to BMD results was observed with some species, which is based on limited numbers of each and trending percents as follows: 13 *C*. *koseri*—46%, 12 *K*. *aerogenes*—67%, 13 *K*. *oxytoca*—38%, 12 *M*. *morganni*—50%. Trending of ComASP (ComASP read) compared to BMD results was observed with lower results for 12 *M*. *morganni* (−42 trending percent) and higher results for 12 *P*. *rettgeri* and 12 *P*. *vulgaris* (50 and 58 trending percent, respectively).

## DISCUSSION

Robust susceptibility testing of antimicrobial agents is important to combat the rise in antimicrobial resistance and encourage appropriate antimicrobial use with the goal of improving patient outcomes. Unfortunately, testing and MIC endpoint reading issues with cefiderocol, particularly for *A. baumannii,* have been reported (11). In addition, the CLSI method for the preparation of iron-depleted CAMHB must be followed closely to bring the final concentration of iron to an acceptable range of <0.03 µg/mL. Thus, CLSI has recommended that laboratories measure residual iron in the medium prior to use (11, 12). Preparation of iron-depleted CAMHB and the requirement to measure residual iron may be beyond the capacity of typical clinical microbiology laboratories. Therefore, an FDA-approved testing device that demonstrates high performance and does not require the preparation of iron-depleted CAMHB could help laboratories implement in-house cefiderocol testing to better inform optimal antimicrobial use by clinicians for multidrug-resistant Gram-negative organisms.

The primary objective of this paper was to describe a multicenter study comparing the ComASP Cefiderocol to CLSI-modified reference broth microdilution for submission to FDA for device clearance. This study was designed to test variables, such as interlaboratory and MIC reading variations, testing reproducibility and detection of resistance with the inclusion of non-susceptible isolates.

The ComASP Cefiderocol device performed well in this multicenter study particularly using the recommended ComASP Cefiderocol reading method (the MIC is read at the first well at which 100% inhibition of growth occurs). Using this reading method for the indicated organisms *A. baumannii* complex, *E. coli, E. cloacae complex, K. pneumoniae, P. mirabilis, P. aeruginosa,* and *S. marcescens*, essential and categorical agreement was >90% for all tested organism except for *E. cloacae* where the categorical agreement was 85.7%. Inter-lab reproducibility was also high with overall reproducibility at 97.4%. Trending was minimal and limited to higher results observed for *P. mirabilis*.

The EA for the consolidated non-clinically indicated species was 95.3% when using the recommended ComASP Cefiderocol reading method. These species were included in the study to provide data to laboratories since the device label is limited to organisms shown to be active clinically and *in vitro* according to the FDA drug-approved label. Although trending was noted for some species, the numbers of each of these species were limited, and further evaluation is warranted.

The inclusion of two reading methods in the study produced interesting results. The cefiderocol CLSI reading method includes two steps. Step 1: read the MIC as the lowest concentration that completely inhibits the organisms’ growth. Step 2: if trailing is observed, read the MIC where growth is significantly reduced compared to the growth control. In this study, two reading methods were employed; CLSI step 2 was applied for those isolates without a clear endpoint (CLSI read), and the ComASP recommended reading method (100% inhibition) was applied to all isolates regardless of the observation of trailing (ComASP read). Interestingly, higher essential and categorical agreement and inter-laboratory reproducibility were observed with the ComASP reading method. Although both the reference BMD and ComASP use round-bottom wells, the design of the ComASP panel differs from the standard 96-well panel in that the diameter of the well is smaller, and each well is surrounded by a second layer of plastic. It is possible that this well design may provide for less visual detection of hazy growth and small buttons of growth that are ignored when reading by the CLSI reference BMD method. The inclusion of one defined reading method in the device package insert should make it easier for the laboratorian to use the device accurately and provide better inter- and intra-laboratory reproducibility.

The ComASP results from four recent method comparison studies performed in Europe were recently published (13–16). These studies were performed prior to the finalization of this 510(k) study and the modified reading guidance. Therefore, it is likely that discrepancies and trending of lower ComASP results reported in some of these studies may be associated with using reading similar to the CLSI method. Other factors that may contribute to differences in the performance are breakpoints utilized, numbers of isolates, and the MIC distribution for the isolates tested and, specifically, the number of isolates near the breakpoint.

In conclusion, the ComASP Cefiderocol performed well in comparison to reference BMD for the species tested. This FDA-cleared device was shown to be a reliable method for testing cefiderocol MIC against relevant clinical isolates and should be a welcome addition to the antibiotic testing method repertoire employed by US clinical microbiology laboratories. These data also highlight the importance of following the IFU of manufacturers exactly. In the case of the ComASP Cefiderocol, there were differences between the two read methods used in the study. The ComASP IFU as cleared by FDA is in accordance with the ComASP read (100% inhibition) as was determined in this study and differs from the CLSI BMD reading methodology.

## SUPPLEMENTAL MATERIAL

Supplemental material jcm.00926-24-s0001.docx Tables S1 and S2; Fig. S1 and S2.

ASM does not own the copyrights to Supplemental Material that may be linked to, or accessed through, an article. The authors have granted ASM a non-exclusive, world-wide license to publish the Supplemental Material files. Please contact the corresponding author directly for reuse.
