# Supplementary material for: A multicenter performance evaluation of cefiderocol MIC results: ComASP in comparison to CLSI broth microdilution
Source: J Clin Microbiol. 2024 Dec 31;63(2):e00926-24. doi: 10.1128/jcm.00926-24 (PMC11837567; doi:10.1128/jcm.00926-24)
Supplement: Supplemental material — Tables S1 and S2; Fig. S1 and S2. [file jcm.00926-24-s0001.docx]

| **Supplemental Table 1 Summary of Study Design** | | | | | | | | | | |
| --- | --- | --- | --- | --- | --- | --- | --- | --- | --- | --- |
| **Total Number and Isolate Category** | ***Number of isolates tested in the study by indicated species*** | | | | | | | **Reference BMD** (one lot of frozen panels; ID-CAMHB made with BD BBL broth); CLSI reading method | **ComASP** (one lot of panels; ID-CAMHB made with BD BBL broth); CLSI and ComASP reading method | **Colony counts** |
|  | ***A. baumannii*** | ***E. cloacae*** | ***E. coli*** | ***K. pneumoniae*** | ***P. mirabilis*** | ***P. aeruginosa*** | ***S. marcescens*** |  |  |  |
| 303 Clinical (Collected at 3 clinical sites)* | 75 | 30 | 33 | 30 | 24 | 90 | 21 | All strains were sent to and tested at LSI | Tested at 3 clinical sites | 10% of isolates/  site |
| 125 Challenge (Includes 55 molecularly characterized isolates) | 17 | 12 | 22 | 20 | 10 | 36 | 8 | Tested at LSI | Tested at LSI on same day as reference BMD using same initial inoculum | 10% of isolates |
| TOTAL  Clinical and Challenge | 92 | 42 | 55 | 50 | 34 | 126 | 29 |  |  |  |
| 10 Reproducibility | 2 |  | 1 | 2 | 1 | 3 | 1 | Not Tested | Tested in triplicate x 3 days x 3 sites (2 clinical sites and Liofilchem site) | One replicate/ day/site for each isolate |
| 2  Quality Control | *E. coli* ATCC 25922 and *P. aeruginosa* ATCC 27853 | | | | | | | Tested at LSI: >20 replicates for EC 25922 and PA 27853 | Tested at 3 clinical sites ≥20 replicates for EC 25922 and PA 27853 with additional testing at LSI and Liofilchem | All replicates / isolate |
| *Non-indicated species were also tested at 3 clinical sites *C. freundii* (n=12), *C. koseri* (n=13), *K. aerogenes* (n=12), *K. oxytoca* (n=17), *M. morganii* (n=12), *P. rettgeri* (n=12), *P. vulgaris* (n=12) | | | | | | | | | | |

| **Supplemental Table 2: Challenge Organism List** | | | | |
| --- | --- | --- | --- | --- |
| **LSI Isolate #** | ***Organism Identification*** *(G. species)* | **BMD MIC** (µg/mL) | **Molecular analysis** |  |
| 6212 | *A. baumannii* | 256 | OXA-23 |  |
| 6213 | *A. baumannii* | 256 |  |  |
| 6214 | *A. baumannii* | 0.5 |  |  |
| 6215 | *A. baumannii* | 0.5 |  |  |
| 6216 | *A. baumannii* | 8 | TEM-1;OXA-23 |  |
| 6860 | *A. baumannii* | >256 |  |  |
| 7030 | *A. baumannii* | 256 |  |  |
| 7330 | *A. baumannii* | 8 |  |  |
| 7681 | *A. baumannii* | 32 |  |  |
| 7899 | *A. baumannii* | 8 |  |  |
| 7951 | *A. baumannii* | 4 |  |  |
| 7980 | *A. baumannii* | 0.5 |  |  |
| 8280 | *A. baumannii* | 16 |  |  |
| 8465 | *A. baumannii* | 16 |  |  |
| 8588 | *A. baumannii* | >256 |  |  |
| 8599 | *A. baumannii* | 32 |  |  |
| 8670 | *A. baumannii* | 8 |  |  |
| 6217 | *E. cloacae* | 8 | TEM-OSBL;NDM-1 |  |
| 6218 | *E. cloacae* | 32 |  |  |
| 6219 | *E. cloacae* | 0.25 |  |  |
| 6221 | *E. cloacae* | 2 |  |  |
| 7337 | *E. cloacae* | 4 |  |  |
| 7538 | *E. cloacae* | 4 |  |  |
| 7539 | *E. cloacae* | 4 |  |  |
| 7721 | *E. cloacae* | 8 |  |  |
| 9236 | *E. cloacae* | 0.06 | ACT-7-like |  |
| 9237 | *E. cloacae* | 16 | ACT-16; SHV-12; TEM-1B |  |
| 9238 | *E. cloacae* | 8 | ACT-Type; OXA-1; SHV-12; TEM-1B; VIM-1 |  |
| 9239 | *E. cloacae* | 8 | ACT-90; SHV-5 |  |
| 6222 | *E. coli* | 4 | CMY-42;CTX-M-15;OXA-181 |  |
| 6223 | *E. coli* | 32 | CTX-M-15;NDM-1;SHV-12;TEM-190 |  |
| 6224 | *E. coli* | 128 | TEM-OSBL;CMY-TYPE |  |
| 6225 | *E. coli* | 8 | TEM-OSBL;CMY-TYPE |  |
| 6227 | *E. coli* | 0.12 |  |  |
| 6228 | *E. coli* | >256 | TEM-1;CTX-M-15;NDM-5 |  |
| 6229 | *E. coli* | 0.12 |  |  |
| 6230 | *E. coli* | 1 |  |  |
| 6231 | *E. coli* | 1 |  |  |
| 6423 | *E. coli* | 4 |  |  |
| 7608 | *E. coli* | 4 |  |  |
| 8452 | *E. coli* | >256 |  |  |
| 8455 | *E. coli* | >256 |  |  |
| 8578 | *E. coli* | 4 |  |  |
| 8608 | *E. coli* | 64 |  |  |
| 8616 | *E. coli* | 2 |  |  |
| 9220 | *E. coli* | 0.5 | CFE-1-like partial CDS; CTX-M-15; OXA-1 |  |
| 9221 | *E. coli* | 64 | CFE-1-like partial CDS; SHV-12; TEM-1B |  |
| 9222 | *E. coli* | 8 | CFE-1-like partial CDS; CTX-M-15 |  |
| 9223 | *E. coli* | >256 | CTX-M-15; NDM-5; OXA-1; TEM-1B |  |
| 9224 | *E. coli* | 4 | EC-Type; OXA-1; OXA-10; TEM-1B; VEB-1 |  |
| 9225 | *E. coli* | 4 | CTX-M-15; EC-Type; OXA-1 |  |
| 6232 | *K. pneumoniae* | 4 | SHV-OSBL;CTX-M-15;NDM-1;OXA-232 |  |
| 6233 | *K. pneumoniae* | 1 |  |  |
| 6234 | *K. pneumoniae* | 128 | TEM-OSBL;SHV-12 |  |
| 6235 | *K. pneumoniae* | 4 | TEM-OSBL;SHV-OSBL;CTX-M-15;OXA-48 |  |
| 6236 | *K. pneumoniae* | 0.03 |  |  |
| 6238 | *K. pneumoniae* | 0.12 |  |  |
| 6239 | *K. pneumoniae* | 0.03 |  |  |
| 6240 | *K. pneumoniae* | 128 | SHV-28;CTX-M-15;NDM-5 |  |
| 6241 | *K. pneumoniae* | 8 |  |  |
| 7230 | *K. pneumoniae* | 2 |  |  |
| 7231 | *K. pneumoniae* | 2 |  |  |
| 7373 | *K. pneumoniae* | 4 |  |  |
| 7669 | *K. pneumoniae* | 4 |  |  |
| 7710 | *K. pneumoniae* | 16 |  |  |
| 8008 | *K. pneumoniae* | 64 |  |  |
| 8263 | *K. pneumoniae* | 256 |  |  |
| 8264 | *K. pneumoniae* | >256 |  |  |
| 9227 | *K. pneumoniae* | 8 | CTX-M-15; LAP-2; OXA-1; OXA-48; SHV-1; TEM-1B |  |
| 9228 | *K. pneumoniae* | 2 | CTX-M-15; OXA-1-Type; SHV-CTX-M-1gr-chimera; TEM-1B |  |
| 9229 | *K. pneumoniae* | 2 | CTX-M-15; KPC-3; OXA-1; OXA-9; SHV-1; TEM-1A |  |
| 787 | *P. aeruginosa* | 0.06 | AmpC |  |
| 3490 | *P. aeruginosa* | 2 | SPM |  |
| 3516 | *P. aeruginosa* | 0.5 | KPC |  |
| 3518 | *P. aeruginosa* | 1 | IMP-14; |  |
| 4393 | *P. aeruginosa* | 1 | GIM-1 |  |
| 4999 | *P. aeruginosa* | 1 | VEB |  |
| 6248 | *P. aeruginosa* | 4 | VIM-45;VEB-1B |  |
| 6249 | *P. aeruginosa* | 2 | PER-1 |  |
| 6250 | *P. aeruginosa* | 1 | VIM-5 |  |
| 6251 | *P. aeruginosa* | 16 | PDC |  |
| 6252 | *P. aeruginosa* | 8 | PER-1;PDC-135 |  |
| 6253 | *P. aeruginosa* | 4 |  |  |
| 6254 | *P. aeruginosa* | 0.06 |  |  |
| 6256 | *P. aeruginosa* | 0.12 |  |  |
| 7529 | *P. aeruginosa* | 128 |  |  |
| 7562 | *P. aeruginosa* | 0.06 |  |  |
| 7565 | *P. aeruginosa* | 16 |  |  |
| 8474 | *P. aeruginosa* | <= 0.016 |  |  |
| 8719 | *P. aeruginosa* | 8 |  |  |
| 8765 | *P. aeruginosa* | 2 |  |  |
| 8768 | *P. aeruginosa* | 16 |  |  |
| 8769 | *P. aeruginosa* | 16 |  |  |
| 8773 | *P. aeruginosa* | 8 |  |  |
| 8774 | *P. aeruginosa* | 8 |  |  |
| 8775 | *P. aeruginosa* | 16 |  |  |
| 8776 | *P. aeruginosa* | 128 |  |  |
| 8778 | *P. aeruginosa* | 0.12 | GES-5 |  |
| 8779 | *P. aeruginosa* | 0.5 | GES-19, GES-20 |  |
| 8786 | *P. aeruginosa* | 2 | IMP-7 |  |
| 8787 | *P. aeruginosa* | 16 | OXA-50-like; PAO-like |  |
| 8794 | *P. aeruginosa* | 1 | IMP-7 |  |
| 8798 | *P. aeruginosa* | 1 | OXA-494-like; PAO-like |  |
| 8799 | *P. aeruginosa* | 2 |  |  |
| 8801 | *P. aeruginosa* | 0.25 | OXA-905; PDC-8 |  |
| 8802 | *P. aeruginosa* | 8 | OXA-395; PDC-30 |  |
| 8803 | *P. aeruginosa* | 4 | AmpC_pae-like, OXA-50-like |  |
| 6242 | *P. mirabilis* | 4 |  |  |
| 6243 | *P. mirabilis* | 16 |  |  |
| 6244 | *P. mirabilis* | 0.5 |  |  |
| 6245 | *P. mirabilis* | <=0.016 |  |  |
| 6246 | *P. mirabilis* | >256 | TEM-2;VEB-6 |  |
| 7668 | *P. mirabilis* | 0.5 |  |  |
| 9210 | *P. mirabilis* | 128 |  |  |
| 9211 | *P. mirabilis* | 16 | TEM-2 |  |
| 9212 | *P. mirabilis* | >256 | TEM-2; VEB-6 |  |
| 9213 | *P. mirabilis* | 128 | OXA-1 |  |
| 8353 | *S. marcescens* | 1 |  |  |
| 9214 | *S. marcescens* | 16 | TEM-10, AmpC_Smar-like |  |
| 9215 | *S. marcescens* | 2 | SHV-12, AmpC_Smar-like |  |
| 9216 | *S. marcescens* | 8 | SHV-12; SRT-2-like |  |
| 9217 | *S. marcescens* | 64 | SRT-2-like |  |
| 9218 | *S. marcescens* | 8 | KPC-2; NDM-1; SHV-12; SRT-2-like; TEM-1B |  |
| 9219 | *S. marcescens* | 2 | CTX-M-3; NDM-5; SRT-2-like |  |
| 9232 | *S. marcescens* | >256 |  |  |

**Supplemental Figure 1. Non-indicated Enterobacterales spp., ComASP Read**

**Supplemental Figure 2. Non-indicated Enterobacterales spp., CLSI Read**
